# Perioperative Coronavirus Disease 2019 Infection and Its Impact on Postoperative Outcomes: Pulmonary Complications and Mortality Based on Korean National Health Insurance Data

**DOI:** 10.3390/jpm15040157

**Published:** 2025-04-17

**Authors:** Hyo Jin Kim, EunJin Ahn, Eun Jung Oh, Si Ra Bang

**Affiliations:** 1 Department of Anesthesiology and Pain Medicine, Chung-Ang University Gwangmyeong Hospital, Chung-Ang University College of Medicine, Gwangmyeong-si 14353, Republic of Korea; raphaella@cau.ac.kr (H.J.K.); aej0226@cauhs.or.kr (E.A.); 2 Department of Anesthesiology and Pain Medicine, Kangbuk Samsung Hospital, Sungkyunkwan University School of Medicine, Seoul 03181, Republic of Korea; ej0156.oh@samsung.com

**Keywords:** COVID-19, mortality rate, perioperative infection, postoperative outcome, pulmonary complication, pandemics

## Abstract

**Background/Objectives**: The coronavirus disease 2019 (COVID-19) pandemic significantly disrupted global healthcare. This study explores the effects of perioperative COVID-19 infection on postoperative outcomes, aiming to refine risk assessment and enhance personalized perioperative care using a comprehensive dataset from the Korean National Health Insurance Service. This analysis extends previous research by providing a large-scale validation of risk factors associated with COVID-19 in a perioperative setting. **Methods**: In this retrospective cohort study, we analyzed data from 2,903,858 patients who underwent surgery under general anesthesia between January 2020 and December 2021. Patients were categorized into COVID-19 (+) and COVID-19 (−) groups within 30 d before or after surgery. Logistic regression models were used to identify independent risk factors for mortality and pulmonary complications. **Results**: After propensity score matching, the final cohort comprised 19,235 patients (COVID-19 (+): 3847; COVID-19 (−): 15,388). The COVID-19 (+) group had significantly higher overall mortality than the COVID-19 (−) group. No significant difference was observed between the groups concerning 30 d mortality. Pulmonary complications, including pneumonia and acute respiratory distress syndrome, were significantly more frequent in the COVID-19 (+) group. The independent predictors of 30 d mortality included advanced age, emergency surgery, and the American Society of Anesthesiologists physical status classification. **Conclusions**: Our study confirms that perioperative COVID-19 infection significantly elevates overall mortality and pulmonary complications, emphasizing the necessity of tailored perioperative management. Incorporating individual risk factors into care protocols not only reduces risks for surgical patients but also enhances treatment approaches. These findings advocate for the implementation of personalized medicine principles in surgical settings to improve patient outcomes during and after the COVID-19 pandemic. This research uses a comprehensive national medical claims dataset to set new standards for studying pandemic health impacts and improving clinical strategies.

## 1. Introduction

The coronavirus disease 2019 (COVID-19) pandemic, declared by the World Health Organization on 11 March 2020, significantly impacted global healthcare systems, posing substantial challenges to surgical care delivery [1]. Patients with perioperative COVID-19 infection are at increased risk of adverse postoperative outcomes, particularly pulmonary complications and mortality [2]. A growing body of evidence supports the correlation between perioperative COVID-19 infection and adverse postoperative outcomes, particularly pulmonary complications and mortality [3]. Previous studies have demonstrated that COVID-19 can trigger a hyperinflammatory response, leading to impaired oxygenation and compromised respiratory function, which increases the likelihood of complications, such as pneumonia, acute respiratory distress syndrome (ARDS), and pulmonary thromboembolism (PTE) [4,5,6,7].

Owing to the COVID-19 pandemic’s transition into an endemic, understanding the clinical impact of perioperative COVID-19 infection remains crucial for optimizing surgical care and preparedness for future public health crises. Even though previous studies have examined the risks of adverse postoperative outcomes in COVID-19-infected patients, large-scale studies are needed to assess these outcomes in diverse populations. Given COVID-19’s profound impact on surgical care, a comprehensive evaluation of mortality and complications among infected patients is essential to inform clinical practice and guide health policy development. As demonstrated by previous studies, population-based research provides crucial evidence for effective policymaking and optimizing healthcare delivery systems [8].

This study hypothesizes that perioperative COVID-19 infection increases overall and 30-day postoperative mortality, and exacerbates pulmonary complications.

The National Health Insurance Service (NHIS) of South Korea provides a unique platform for analyzing nationwide data to assess the impact of perioperative COVID-19 infection [9]. Unlike institution-specific datasets, the NHIS includes nearly the entire population, enabling a thorough evaluation of surgical outcomes during the pandemic [10]. This extensive dataset allows for the assessment of both short-term (30 d) and long-term (overall) mortality, as well as the burden of pulmonary and systemic complications.

In this study, we aimed to examine the impact of perioperative COVID-19 infection on postoperative outcomes, focusing on overall mortality, 30 d mortality, and pulmonary complications. By analyzing large-scale, nationwide data from the NHIS, we aimed to provide evidence to inform perioperative management strategies and guide health policy decisions during and after the COVID-19 pandemic. This analysis provides a foundation for developing individualized medical strategies based on specific risk profiles identified in our data. Implementing such tailored approaches is essential for improving surgical outcomes and enhancing patient safety in the context of ongoing health crises.

## 2. Materials and Methods

### 2.1. Study Design

In this study, we employed a retrospective cohort design using de-identified healthcare claims data from the Korean NHIS. The NHIS provides universal coverage to nearly the entire Korean population, making its dataset a valuable resource for population-level health studies. Healthcare providers submit claims for reimbursement to the NHIS, and these claims undergo review by the Health Insurance Review and Assessment Service. This study’s dataset was derived from the National Health Information Database (NHID) maintained by the NHIS. The NHID contains comprehensive information, including information about healthcare utilization, health screening results, sociodemographic data, and mortality records. Researchers are granted access to the NHID upon approval of their study protocols by the NHIS review committee, ensuring compliance with ethical and security standards [11]. Healthcare claims data comprise detailed information, such as patient demographics, medical institutions, diagnoses coded using the International Classification of Diseases, 10th revision (ICD-10), procedures, prescriptions, and costs. Researchers are granted access to customized datasets that meet pre-specified study conditions; however, the raw data remain inaccessible. Data analysis can be conducted at designated analysis centers or, in certain cases, via secure remote access systems [10]. This study’s protocol was reviewed and approved by the Institutional Review Board of Inje University Seoul Paik Hospital (PAIK 2022-09-005) on 15 September 2022. The requirement for informed consent was waived owing to the use of de-identified administrative data.

### 2.2. Study Population

This study included all patients who underwent surgery under general anesthesia in hospitals across Korea between 1 January 2020 and 31 December 2021. The inclusion criterion was admission for surgery coded with specific general anesthesia codes (L0101, L1211, L1221, L1212, L1222). Patients with a diagnosis of pneumonia (ICD-10 codes J12–J18) recorded within 1 year before surgery were excluded to reduce potential confounding effects related to pre-existing respiratory conditions.

Patients were categorized into the COVID-19 (+) group, comprising patients with a confirmed COVID-19 diagnosis (ICD-10 code U07.1) within 30 d before or after surgery, and the COVID-19 (−) group, comprising patients without such a diagnosis. Propensity score matching (PSM) was performed to balance baseline characteristics, including age, sex, comorbidities, American Society of Anesthesiologists (ASA) classification, and hospital type, ensuring comparability between the groups for subsequent analyses.

### 2.3. Variables and Outcomes

Demographic variables included age, sex, economic status, and residential region. The recorded surgical details were emergency surgery status and nighttime surgery, which was defined as surgeries starting between 6:00 p.m. and 8:00 a.m. The hospital type was categorized into clinics, hospitals, general hospitals, and tertiary hospitals based on the number of beds and specialization criteria. Comorbidities were quantified using the Charlson Comorbidity Index (CCI) and Elixhauser Comorbidity Score, with ICD-10 codes. The major comorbidities included cardiovascular diseases (congestive heart failure, arrhythmias, and hypertension), cerebrovascular diseases (including stroke and transient ischemic attacks), chronic pulmonary diseases, diabetes (with or without complications), renal failure, liver disease, and malignancies (including metastatic cancer). Moreover, additional conditions, such as obesity, coagulopathy, and psychiatric disorders (such as depression and psychoses), were considered.

Hospitals were categorized by capacity and service level: clinics with up to 29 inpatient beds, hospitals with a minimum of 30 beds, general hospitals with at least 100 beds and physician specialists, and tertiary hospitals providing comprehensive advanced care across at least 20 departments [12].

This study’s primary outcomes were overall mortality, defined as death from any cause during the follow-up period, and 30 d mortality, defined as death within 30 d of surgery. Secondary outcomes included pulmonary complications (pneumonia, ARDS, PTE, and unplanned mechanical ventilation), intensive care unit (ICU) admissions, ICU length of stay, cardiac arrest, myocardial infarction, venous thromboembolic events, surgical site infections, sepsis, acute renal failure, hepatic failure, and overall length of hospital stay.

### 2.4. Statistical Analysis

To minimize selection bias and balance baseline characteristics between the perioperative COVID-19 (+) and COVID-19 (−) groups, a 1:4 PSM was performed using a greedy nearest-neighbor matching method. Propensity scores were estimated using logistic regression, incorporating age (modeled as a continuous variable), CCI, ASA physical status classification (≥3), and comorbidities, including hypertension, diabetes, liver disease, and kidney disease. A caliper width of 0.1 times the standard deviation of the logit-transformed propensity score (logit propensity score) was applied to ensure closely matched pairs. Matching quality was assessed using standardized mean differences, with values <0.1 indicating acceptable balance. After matching, the final study cohort comprised 3847 patients in the COVID-19 (+) group and 15,388 patients in the COVID-19 (−) group, totaling 19,235 matched patients.

Given the comprehensive coverage of the NHIS dataset, traditional sample size calculations typically used in prospective studies to estimate power were not performed. This approach is aligned with studies utilizing large, comprehensive registries where the sample includes a substantial portion or the entirety of a population, thus providing maximal statistical power inherent to the dataset size [13].

Baseline characteristics were summarized using means and standard deviations for continuous variables and frequencies and percentages for categorical variables. The normality of continuous variables was evaluated using the Kolmogorov–Smirnov or Shapiro–Wilk test. Regarding prematching data, continuous variables were compared using the Wilcoxon rank-sum test, whereas categorical variables were analyzed using chi-square or Fisher’s exact tests. After matching, continuous variables were compared using paired t-tests or Wilcoxon signed-rank tests, and categorical variables were analyzed using McNemar or exact McNemar tests. Additionally, multivariable logistic regression models were employed to evaluate the association between perioperative COVID-19 infection and outcomes, with adjustments made for relevant covariates. Adjusted odds ratios (ORs) with 95% confidence intervals (CIs) were calculated for all regression models. All statistical tests were two-sided, with a *p*-value < 0.05 considered statistically significant. Statistical analyses were conducted using R software (version 4.2.2) and SAS Enterprise Guide (version 6.1; SAS Institute Inc., Cary, NC, USA).

## 3. Results

**Figure 1:**
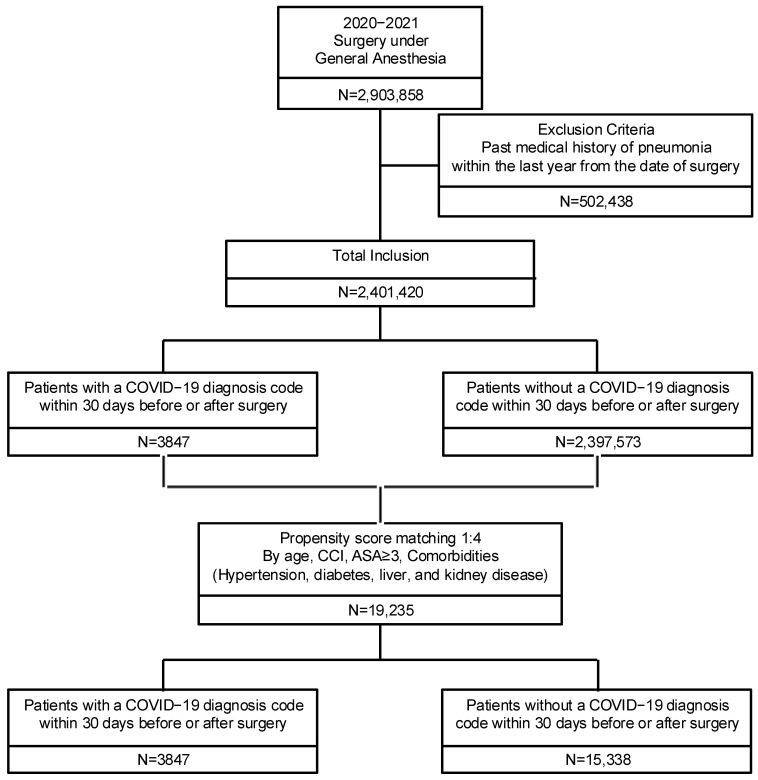
Flow diagram of the study population.

In Korea, 2,903,858 patients underwent surgery under general anesthesia between 2020 and 2021. Of these, 502,438 were excluded from this study owing to the presence of a pneumonia diagnosis code recorded within 1 year before undergoing surgery. Consequently, 2,401,420 patients were included in this study’s analysis. Following PSM, the final analysis comprised 3847 patients in the COVID-19 (+) group and 15,388 in the COVID-19 (−) group (Figure 1).

Table 1 presents the summarized baseline characteristics of patients in the prematching and postmatching cohorts. Regarding the prematching cohort, 2,401,420 patients were analyzed, including 3847 (0.16%) in the COVID-19 (+) group and 2,397,573 (99.84%) in the COVID-19 (−) group. Significant differences were observed between the two groups across several variables, including year of surgery, sex, age, hospital type, hospitalization path, ASA classification, and the CCI. Patients in the COVID-19 (+) group had higher proportions of ASA classification of ≥3, more frequent nighttime surgeries, and higher CCI values than those in the COVID-19 (−) group. Most patients in both groups underwent surgery in 2021, were female, were aged between 19 and 69 years, and were treated at tertiary hospitals. After PSM, a total of 19,235 patients were included in the analysis, with 3847 in the COVID-19 (+) group and 15,388 in the COVID-19 (−) group.

Table 2 presents the postoperative outcomes and complications of patients who underwent surgery under general anesthesia, comparing the COVID-19 (+) and COVID-19 (−) groups before and after PSM. Post-matching, the overall mortality rate was higher in the COVID-19 (+) group than that in the COVID-19 (−) group (6.20% vs. 5.93%, *p* = 0.002). The 30 d postoperative mortality rates were 0.86% in the COVID-19 (+) group and 0.70% in the COVID-19 (−) group, with no statistically significant difference (*p* = 0.31). Additionally, the time to death was shorter in the COVID-19 (+) group than that in the COVID-19 (−) group (220.9 d vs. 311.4 d, *p* < 0.0001).

Regarding postoperative complications, the COVID-19 (+) group demonstrated significantly higher incidences of pneumonia, ARDS, PTE, and surgical site infections in both the prematching and postmatching cohorts (*p* < 0.05). Moreover, thromboembolic events, ventilator requirement, acute kidney injury, and hepatic failure were more frequent in the COVID-19 (+) group before matching (*p* < 0.05); however, these differences were no longer statistically significant following PSM (*p* > 0.05). Furthermore, ICU admissions were more frequent among the COVID-19 (+) group than those among the COVID-19 (−) group (14.79% vs. 11.31%, *p* < 0.0001); however, the duration of ICU stays was similar between the groups.

**Figure 2:**
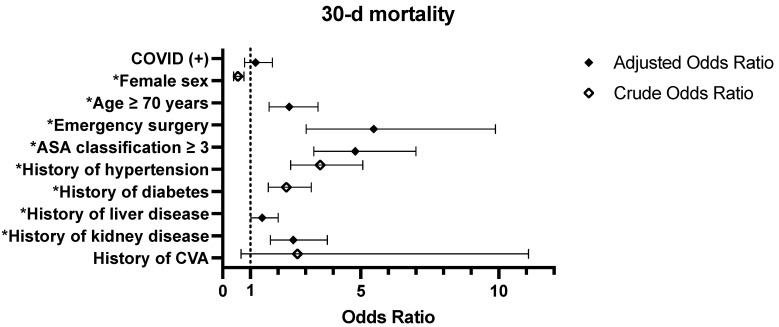
Odds ratios for 30-day mortality related to COVID-19 infection in surgical patients. Crude and adjusted odds ratios for factors influencing 30-day mortality among surgical patients are displayed. Crude odds ratios are calculated relative to COVID-19-negative patients. Adjusted odds ratios consider additional covariates such as age, emergency surgery status, ASA score, and kidney disease. Error bars represent 95% confidence intervals. Statistically significant values (*p* < 0.05) are marked with an asterisk (*). The dashed vertical line at odds ratio = 1.0 indicates the null value, representing no difference between groups.

Regarding the univariable analysis, the crude OR for 30 d mortality in the COVID-19 (+) group was 1.224 (95% CI: [0.828–1.811], *p* = 0.311), which was not statistically significant (Table 3). Further details are visually represented in Figure 2. Regarding the multivariable analysis, the risk of 30 d mortality was significantly higher among specific subgroups. Patients aged ≥70 years had an adjusted OR of 2.407 (95% CI: [1.680–3.449], *p* < 0.0001), those who underwent emergency surgery had an adjusted OR of 5.468 (95% CI: [3.026–9.881], *p* < 0.001), patients with an ASA score of ≥3 had an adjusted OR of 4.803 (95% CI: [3.297–6.997], *p* < 0.001), and individuals with kidney disease had an adjusted OR of 2.555 (95% CI: [1.724–3.786], *p* < 0.001).

**Figure 3:**
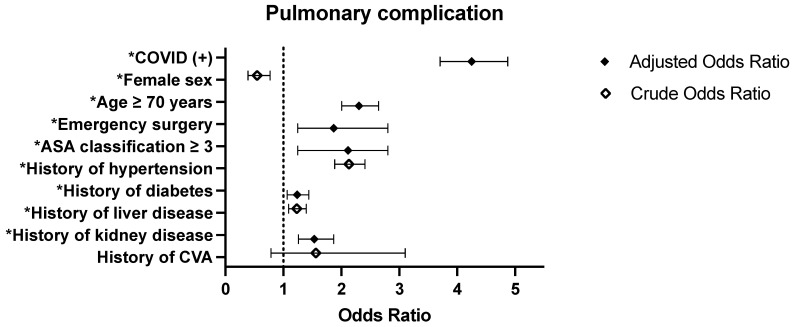
Odd ratios for the risk of pulmonary complications in surgical patients with and without COVID-19 infection. Crude and adjusted odds ratios for factors influencing the risk of pneumonia among surgical patients are displayed. Crude odds ratios are calculated relative to COVID-19 negative patients. Adjusted odds ratios account for additional covariates such as age, emergency surgery status, ASA score, kidney disease, and diabetes. Error bars represent 95% confidence intervals. Statistically significant values (*p* < 0.05) are marked with an asterisk (*). The dashed vertical line at odds ratio = 1.0 indicates the null value, representing no difference between groups.

The COVID-19 (+) group had a significantly higher risk of developing pneumonia than the COVID-19 (−) group (adjusted OR: 4.250, 95% CI: [3.704–4.875], *p* < 0.0001), as shown in Table 4 and Figure 3. Other independent predictors of pneumonia included age ≥70 years, emergency surgery, and an ASA score of ≥3. Additionally, patients with a history of kidney disease were at a significantly higher risk of pneumonia. Furthermore, patients with a history of diabetes also exhibited an increased risk of pneumonia. Conversely, female patients exhibited a reduced risk of pneumonia.

## 4. Discussion

In this study, we examined the impact of COVID-19 infection on postoperative outcomes in patients who underwent surgery under general anesthesia during the COVID-19 pandemic. By analyzing nearly 3 million patients from the Korean NHIS dataset, this study identified significant differences in mortality rates, pulmonary complications, ICU admission rate, and other risk factors between COVID-19 (+) and COVID-19 (−) groups, even after adjusting for baseline characteristics using PSM.

From an epidemiological perspective, this study’s findings revealed notable trends. Compared with 2020, the increased number of surgeries performed in 2021 likely reflects the healthcare system’s recovery and adaptation, including the resumption of elective procedures after the initial pandemic disruptions. This trend emphasizes the healthcare infrastructure’s ability to respond and stabilize after a global health crisis [14]. Additionally, the higher proportion of surgeries performed on female patients and the predominance of patients aged 18–69 years suggest a demographic pattern where younger and healthier individuals were prioritized for surgical intervention. Conversely, older adults (aged ≥ 70 years) and pediatric populations underwent fewer procedures, potentially reflecting risk mitigation strategies and a focus on urgent or essential care during the pandemic [15]. Furthermore, this study’s findings indicated that the COVID-19 (+) group tended to have a higher ASA classification (ASA 3 or higher), were more likely to undergo nighttime surgeries, and had a higher incidence of comorbid conditions than the COVID-19 (−) group. These findings suggest that COVID-19-infected patients who required surgery were generally sicker and required more urgent and complex care [16,17,18].

After matching, the COVID-19 (+) group exhibited higher mortality rates for both overall (6.20% vs. 5.93%, *p* = 0.002) and 30 d postoperative mortality (0.86% vs. 0.70%, *p* = 0.31). The 30 d mortality rates did not reach statistical significance; however, the overall trend indicated worse outcomes for the COVID-19 (+) group. These findings align with those of previous studies that reported increased mortality and pulmonary complications in surgical patients with COVID-19 infection [3,19,20]. In this study, we defined the COVID-19 (+) group as patients assigned a COVID-19 diagnosis code (U071) within 30 d before or after surgery. This 30 d window, supported by previous studies, reflected the typical recovery period for acute COVID-19 and allowed for a comprehensive assessment of its short-term impact on surgical outcomes [21,22,23]. The shorter time to death observed among the COVID-19 (+) group suggested a more rapid postoperative clinical deterioration. This finding emphasizes the importance of preoperative COVID-19 screening and careful perioperative management to mitigate risks among infected patients. Similarly, Prasad et al. [24] found significantly lower survival probabilities among COVID-19-infected patients using Kaplan–Meier analyses, consistent with this study’s findings of a steeper decline in survival curves among the COVID-19 (+) group than that in the COVID-19 (−) group.

Pulmonary complications, a major concern following general anesthesia, were significantly more prevalent among the COVID-19 (+) group than in the COVID-19 (−) group; these complications included pneumonia, ARDS, and PTE. The need for mechanical ventilation was initially higher in the COVID-19 (+) group; however, this difference was no longer statistically significant after PSM. The increased risk of pulmonary complications may be attributed to the distinct pathophysiological mechanisms of COVID-19, such as endothelial damage, microvascular thrombosis, and impaired gas exchange [25]. The severe acute respiratory syndrome coronavirus-2 infection triggers an exaggerated inflammatory response, leading to cytokine storms and diffuse alveolar damage that impair pulmonary function and increase susceptibility to ARDS [26,27]. Furthermore, COVID-19-associated hypercoagulability and immunothrombosis promote microvascular obstruction within the pulmonary vasculature, leading to ventilation–perfusion mismatch and exacerbating hypoxemia [28,29]. The virus interacts with ACE2 receptors on pneumocytes, macrophages, and endothelial cells, disrupting pulmonary architecture and inducing a pro-inflammatory state that increases vascular permeability and tissue damage [30,31]. Additionally, the binding of the virus to endothelial ACE2 receptors triggers systemic effects, including severe respiratory impairment from ARDS, leading to hypoxia. This hypoxia, combined with a potent inflammatory response marked by elevated cytokines like IL-1, IL-6, and TNF-α, results in oxygen supply–demand mismatches, further complicating the clinical picture.

Multivariable logistic regression analysis identified COVID-19 infection as a significant independent risk factor for postoperative pneumonia, with an adjusted OR of 3.62. This elevated risk is consistent with the known respiratory pathophysiology of COVID-19, characterized by systemic inflammation, diffuse lung injury, and immune dysfunction. Other significant predictors of postoperative pneumonia included advanced age (≥70 years), emergency surgery, and higher ASA classification (≥3), which were associated with reduced physiological reserves and impaired recovery capacity [32]. Additionally, pre-existing kidney disease and diabetes were identified as significant risk factors, further emphasizing the role of comorbidities in increasing vulnerability to pulmonary complications [33]. Diabetes contributes to hyperglycemia-induced immune dysfunction, increased viral replication, and chronic inflammation, which collectively exacerbate respiratory and systemic complications [34]. Similarly, chronic kidney disease (CKD) is associated with a compromised immune response and heightened susceptibility to infections, further amplifying the risk of pulmonary complications [35]. Notably, female sex was identified as a protective factor, which may be attributed to estrogen’s immunomodulatory effects and its role in enhancing immune response, as well as reducing severe inflammatory processes [36]. These findings provide evidence for the need for proactive respiratory care and vigilant monitoring, particularly among high-risk patients with COVID-19 or significant comorbidities [37].

This study’s analysis of 30 d mortality risk factors demonstrated the critical influence of age, emergency surgery, ASA classification, and pre-existing comorbidities on postoperative outcomes. Patients aged ≥70 years exhibited a 2.26-fold higher mortality risk, reflecting the well-established association between advanced age and reduced physiological resilience [38]. Emergency surgery was associated with a 5.69-fold increased risk of 30 d mortality, reflecting the critical challenges posed by acute clinical deterioration and limited time for preoperative optimization. Brown et al. [39] reported that patients undergoing emergency surgery with concurrent COVID-19 infection had a perioperative mortality odds ratio of 7.9. In addition, patients with ASA classification 3 or higher exhibited a 4.52-fold increased risk of 30 d mortality. This finding aligns with previous studies that identified higher ASA scores as a strong predictor of postoperative mortality, particularly among patients with multiple comorbidities [40]. Moreover, this study revealed that pre-existing kidney disease was associated with a 2.16-fold increased risk of 30 d mortality, which was consistent with the findings of previous studies that reported CKD as a significant risk factor for COVID-19-related mortality. Gansevoort et al. [41] reported that patients with severe CKD, particularly those with an estimated glomerular filtration rate below 30 mL/min/1.73 m^2^ or those undergoing dialysis, exhibited a markedly elevated mortality risk, exceeding that of other comorbid conditions, such as chronic heart or lung disease. Renal dysfunction exacerbates systemic inflammation, compromises hemodynamic stability, and contributes to multiorgan failure, further increasing surgical vulnerability among affected patients [42].

The substantial increase in mortality and pulmonary complications in the COVID-19 (+) group demonstrated the need for targeted perioperative care strategies. Comprehensive preoperative assessments, including respiratory and functional evaluations, are particularly important for high-risk patients, such as those of advanced age, those undergoing emergency surgery, or those with significant comorbidities, such as CKD. Intraoperative and postoperative management should include advanced respiratory support, thromboprophylaxis, and strict infection control protocols to reduce adverse outcomes [43,44]. These results provide practical evidence to improve perioperative management during infectious disease outbreaks. Effective preoperative screening and improved care protocols are crucial to reduce risks for surgical patients with COVID-19. These measures can help identify and manage high-risk patients effectively, potentially reducing the incidence of severe complications [31]. Additionally, examining outcomes across different countries could highlight valuable lessons, as variations in healthcare systems and population demographics may influence the effectiveness of perioperative strategies [45,46].

This study has several strengths. First, it utilized a large-scale national dataset, including nearly three million patients, enabling a comprehensive evaluation of perioperative outcomes in a real-world setting. Second, the application of PSM minimized baseline differences between the COVID-19 (+) and COVID-19 (−) groups, improving the validity of the comparisons. Third, the study provides detailed analyses of mortality and pulmonary complications, offering valuable insights into COVID-19 infection’s impact on surgical patients.

However, this study has some limitations. First, the data used in this study were derived from health insurance claims rather than electronic medical records, which may have limitations concerning clinical detail and accuracy for research purposes. Claims data are predominantly used for administrative and billing purposes; therefore, they may not capture the full spectrum of clinical information, such as detailed laboratory findings or imaging results. Second, the analysis was restricted to the COVID-19 pandemic period (2020–2021), preventing comparisons with pre-pandemic surgical outcomes. Third, this study did not account for key factors, such as COVID-19 variants, vaccination status, or changes in treatment protocols, which may have influenced patient outcomes. Future prospective studies incorporating these variables and assessing long-term outcomes are essential to confirm and expand this study’s findings. Moreover, while this study provides insights specific to the South Korean context during the pandemic, the findings may not fully apply to other settings due to variations in healthcare systems and patient demographics. Caution should be exercised when generalizing these results internationally. Additionally, while propensity score matching helped control observable confounders, the potential for residual confounding by unmeasured variables remains.

## 5. Conclusions

In this study, we examined perioperative COVID-19 infection’s impact on postoperative outcomes using Korean NHIS data. COVID-19 infection was associated with significantly higher overall mortality and increased pulmonary complications, such as pneumonia, ARDS, and PTE. Despite 30 d postoperative mortality not reaching statistical significance after matching, a trend toward worse outcomes in the COVID-19 (+) group remained. Advanced age, emergency surgery, higher ASA classification, and pre-existing CKD were identified as significant risk factors for both 30 d mortality and pulmonary complications. Consequently, integrating personalized medicine into perioperative management, based on identified risk factors such as age, emergency status, and comorbid conditions, is essential for improving outcomes for COVID-19-affected surgical patients. Future research should explore the impact of COVID-19 variants and vaccination status on surgical outcomes to further refine these strategies and enhance patient care in the post-pandemic era.

## Figures and Tables

**Table 1:** Baseline characteristics before and after propensity score matching.

Variable	Prematching					Postmatching				
	Total (N = 2,401,420)	COVID (+) (N = 3847)	COVID (−) (N = 2,397,573)	*p*-Value	SMD	Total (N = 19,235)	COVID (+) (N = 3847)	COVID (−) (N = 15,388)	*p*-Value	SMD
Year										
2020	1,200,631 (50)	759 (19.73)	1,199,872 (50.05)	<0.0001		8308 (43.19)	759 (19.73)	7549 (49.06)	<0.0001	
2021	1,200,789 (50)	3088 (80.27)	1,197,701 (49.95)			10,927 (56.81)	3088 (80.27)	7839 (50.94)		
Sex										
Male	1,013,593 (42.21)	1780 (46.27)	1,011,813 (42.2)	<0.0001	0.1463	8898 (46.26)	1780 (46.27)	7118 (46.26)	0.9885	0.00026
Female	1,387,827 (57.79)	2067 (53.73)	1,385,760 (57.8)			10,337 (53.74)	2067 (53.73)	8270 (53.74)		
Age										
<18 years	154,092 (6.42)	240 (6.24)	153,852 (6.42)	<0.0001	0.3812	1201 (6.24)	240 (6.24)	961 (6.25)	0.9999	0.00022
18–69 years	1,830,632 (76.23)	2737 (71.15)	1,827,895 (76.24)			13,685 (71.15)	2737 (71.15)	10,948 (71.15)		
≥70 years	416,696 (17.35)	870 (22.62)	415,826 (17.34)			4349 (22.61)	870 (22.62)	3479 (22.61)		
Hospital type										
Tertiary Hospital	840,699 (35.01)	1978 (51.42)	838,721 (34.98)	<0.0001		8032 (41.76)	1978 (51.42)	6054 (39.34)	<0.0001	
General Hospital	787,954 (32.81)	1125 (29.24)	786,829 (32.82)			6245 (32.47)	1125 (29.24)	5120 (33.27)		
Hospital	515,418 (21.46)	507 (13.18)	514,911 (21.48)			3428 (17.82)	507 (13.18)	2921 (18.98)		
Clinic	257,349 (10.72)	237 (6.16)	257,112 (10.72)			1530 (7.95)	237 (6.16)	1293 (8.4)		
Hospitalization Pathway										
Emergency	289,143 (12.04)	518 (13.47)	242,904 (10.13)	<0.0001		1379 (7.17)	518 (13.47)	1893 (12.3)	0.0008	
Outpatient	1,869,146 (77.84)	3102 (80.63)	288,625 (12.04)			2411 (12.53)	3102 (80.63)	12,343 (80.21)		
Others	243,131 (10.12)	227 (5.9)	1,866,044 (77.83)			15,445 (80.3)	227 (5.9)	1152 (7.49)		
Emergency surgery	31,493 (1.31)	44 (1.14)	31,449 (1.31)	0.3602		274 (1.42)	44 (1.14)	230 (1.49)	0.1004	
Night surgery	61,790 (2.57)	127 (3.3)	61,663 (2.57)	0.0043		542 (2.82)	127 (3.3)	415 (2.7)	0.0428	
ASA 3 or higher	237,558 (9.89)	562 (14.61)	236,996 (9.88)	<0.0001		2628 (13.66)	562 (14.61)	2066 (13.43)	0.0561	
Comorbidities										
History of Hypertension	776,749 (32.35)	1494 (38.84)	775,255 (32.33)	<0.0001		7742 (40.25)	1494 (38.84)	6248 (40.6)	0.0455	
History of Diabetes	466,756 (19.44)	972 (25.27)	465,784 (19.43)	<0.0001		5347 (27.8)	972 (25.27)	4375 (28.43)	<0.0001	
History of liver disease	680,836 (28.35)	1461 (37.98)	679,375 (28.34)	<0.0001		7136 (37.1)	1461 (37.98)	5675 (36.88)	0.2072	
History of kidney disease	87,212 (3.63)	231 (6)	86,981 (3.63)	<0.0001		1228 (6.38)	231 (6)	997 (6.48)	0.2817	
History of CVA	8327 (0.35)	13 (0.34)	8314 (0.35)	0.9257		103 (0.54)	13 (0.34)	90 (0.58)	0.0605	
Charson Comorbidity Index										
Continuous	1.91 (1.51–2.38)	1.95 (1.59–2.59)	1.91 (1.51–2.38)	0.0471	0.010393	1.86 (1.59–2.52)	1.9513 (1.59–2.59)	1.863855 (1.60–2.52)	1	0.00033
0	814,029 (33.9)	855 (22.23)	813,174 (33.92)	<0.0001		4275 (22.23)	855 (22.23)	3420 (22.23)	1	
1	512,184 (21.33)	665 (17.29)	511,519 (21.33)			3325 (17.29)	665 (17.29)	2660 (17.29)		
2	374,303 (15.59)	558 (14.5)	373,745 (15.59)			2790 (14.5)	558 (14.5)	2232 (14.5)		
3+	700,904 (29.19)	1769 (45.98)	699,135 (29.16)			8845 (45.98)	1769 (45.98)	7076 (45.98)		
Elixhauser’s comorbidities										
Weight (Continuous)	4.68 (2.29–7.69)	5.64 (3.53–9.16)	4.68 (2.28–7.69)	<0.0001		5.03 (3.20–8.25)	5.64 (3.52–9.16)	4.88 (3.12–7.99)	<0.0001	

Values are expressed as absolute numbers (percentages) for categorical variables. Values are presented as mean (standard deviation) for continuous variables that are normally distributed and median (interquartile range) for continuous variables that are not normally distributed. SMD—standardized mean difference; ASA—American Society of Anesthesiologists; CVA—cerebrovascular accidents.

**Table 2:** Postoperative outcomes and complications before and after propensity score matching.

Variable	Prematching				Postmatching			
	Total (N = 2,401,420)	COVID (+) (N = 3847)	COVID (−) (N = 2,397,573)	*p*-Value	Total (N = 19,235)	COVID (+) (N = 3847)	COVID (−) (N = 15,388)	*p*-Value
Overall mortality	92,734 (3.86)	280 (7.28)	92,454 (3.86)	<0.0001 *	1193 (6.20)	280 (7.28)	913 (5.93)	0.002 *
30 d mortality	11,709 (0.49)	33 (0.86)	11,709 (0.49)	0.001 *	141 (0.73)	33 (0.86)	108 (0.70)	0.3104
Time to death (days)	305.2 (167.9)	220.9 (155.9)	305.5 (168.0)	<0.0001 *	290.2 (157.5)	220.9 (155.9)	311.4 (158.2)	<0.0001 *
Postoperative complication								
Pneumonia	73,402 (3.06)	481 (12.5)	72,921 (3.04)	<0.0001 *	1097 (5.7)	481 (12.5)	616 (4)	<0.0001 *
ARDS	1774 (0.07)	33 (0.86)	1741 (0.07)	<0.0001 *	41 (0.21)	33 (0.86)	8 (0.05)	<0.0001 *
PTE	17,452 (0.73)	67 (1.74)	17,385 (0.73)	<0.0001 *	194 (1.01)	67 (1.74)	127 (0.83)	<0.0001 *
Thromboembolic event	18,333 (0.76)	40 (1.04)	18,293 (0.76)	0.0487 *	197 (1.02)	40 (1.04)	157 (1.02)	0.9145
Mechanical ventilation	1177 (0.05)	5 (0.13)	1172 (0.05)	0.0232 *	12 (0.06)	5 (0.13)	7 (0.05)	0.0605
Cardiac arrest	8300 (0.35)	14 (0.36)	8286 (0.35)	0.8466	89 (0.46)	14 (0.36)	75 (0.49)	0.3128
MI	23,132 (0.96)	40 (1.04)	23,092 (0.96)	0.6268	265 (1.38)	40 (1.04)	225 (1.46)	0.0444
Surgical site infection	66,537 (2.77)	258 (6.71)	66,279 (2.76)	<0.0001 *	766 (3.98)	258 (6.71)	508 (3.3)	<0.0001 *
Sepsis	37,158 (1.55)	123 (3.2)	37,035 (1.54)	<0.0001 *	492 (2.56)	123 (3.2)	369 (2.4)	0.005 *
ARF	33,711 (1.4)	72 (1.87)	33,639 (1.4)	0.0136 *	386 (2.01)	72 (1.87)	314 (2.04)	0.5039
Hepatic failure	8392 (0.35)	32 (0.83)	8360 (0.35)	<0.0001 *	124 (0.64)	32 (0.83)	92 (0.6)	0.1049
Hospitalization								
Hospitalization	2,241,214 (93.38)	3739 (90.30)	2,237,475 (93.32)		18,388 (95.60)	3739 (90.30)	14,649 (95.20)	
Hospital length of stay	21.8 (14.7–52.3)	32.3 (10.0–63.7)	21.7 (14.7–52.2)	<0.0001 *	24.4 (13.2–55.9)	32.3 (10.00–63.7)	22.4 (14.0–53.6)	<0.0001 *
Hospitalization costs	11,867 (5933–24,562)	7473 (3345–17,618)	11,857 (5931–24,544)	<0.0001 *	13,499 (6887–27,580)	17,619 (7473–33,452)	12,452 (6737–25,773)	<0.0001 *
ICU Admission								
ICU admission	213,008 (8.87)	569 (14.79)	212,439 (8.86)	<0.0001 *	2310 (12.01)	569 (14.79)	1741 (11.31)	<0.0001 *
ICU length of stay	72.2 (17.8–245.6)	156.0 (17.7–1487.6)	72.0 (17.8–236.0)	0.9038	89.9 (17.8–746.1)	156.0 (17.7–1487.6)	69.3 (17.8–190.4)	0.9415
ICU costs	76,450 (19,885–238,958)	103,490 (20,067–1,003,210)	76,322 (19,885–234,360)	0.8133	88,293 (19,998–523,600)	103,490 (20,067–136,210)	73,246 (19,976–154,000)	0.928
ECMO implementation	18,360 (0.76)	29 (0.75)	18,331 (0.76)	0.9391	158 (0.82)	29 (0.75)	129 (0.84)	0.6036

Values are expressed as absolute numbers (percentages) for categorical variables. Values are presented as the mean (standard deviation) for continuous variables that are normally distributed and median (interquartile range) for continuous variables that are not normally distributed. * *p* < 0.05 indicates statistically significant differences between groups. All costs are presented in thousands of KRW. ARDS—acute respiratory distress syndrome; PTE—pulmonary thromboembolism; MI—myocardial infarction; ARF—acute renal failure; ICU—intensive care unit; ECMO—Extracorporeal Membrane Oxygenation.

**Table 3:** Logistic regression analysis for 30 d mortality in the COVID-19 (+) group compared with that in the matched COVID-19 (−) group.

	Univariable		Multivariable	
	Crude OR (95% CI)	*p*-Value	Adjusted OR (95% CI)	*p*-Value
COVID Group				
COVID (–)	1		1	
COVID (+)	1.224 (0.828–1.811)	0.311	1.189 (0.788–1.794)	0.4087
Year				
2020	1			
2021	1.057 (0.756–1.479)	0.7457		
Sex				
Male	1		1	
Female	0.548 (0.39–0.77)	0.0005		
Age				
<70 years	1		1	
≥70 years	4.974 (3.551–6.969)	<0.0001	2.407 (1.68–3.449)	<0.0001 *
Hospital type				
Tertiary care hospital	1		1	
General hospital	1.45 (1.036–2.028)	<0.0001	1.46 (1.037–2.055)	0.0008 *
Hospital	0.072 (0.018–0.292)	0.0143	0.167 (0.04–0.692)	0.0667
Clinic	0.08 (0.011–0.578)	0.0858	0.242 (0.033–1.775)	0.3595
Emergency surgery	7.985 (4.537–14.055)	<0.0001	5.468 (3.026–9.881)	<0.0001 *
ASA 3 or higher	10.821 (7.677–15.254)	<0.0001	4.803 (3.297–6.997)	<0.0001 *
Comorbidities				
History of hypertension	3.531 (2.459–5.072)	<0.0001		
History of diabetes	2.302 (1.651–3.209)	<0.0001		
History of liver disease	1.829 (1.313–2.548)	0.0004	1.429 (1.019–2.005)	0.0386 *
History of kidney disease	5.969 (4.12–8.649)	<0.0001	2.555 (1.724–3.786)	<0.0001 *
History of CVA	2.706 (0.661–11.077)	0.1663		
Charson Comorbidity Index				
0	1			
1	1.674 (0.733–3.822)	0.3033		
2	2.46 (1.115–5.429)	0.5035		
3+	4.976 (2.596–9.536)	<0.0001		
Elixhauser’s comorbidities				
Weight(Continuous)	1.26 (1.201–1.321)	<0.0001		

Values are presented as odds ratios with corresponding 95% confidence intervals. * *p* < 0.05 indicates statistically significant differences between groups. OR—odds ratio; CI—confidence interval; ASA—American Society of Anesthesiologists.

**Table 4:** Logistic regression analysis for overall pulmonary complications in the COVID (+) group compared with that in the matched COVID (−) group.

	Univariable		Multivariable	
	Crude OR (95% CI)	*p*-Value	Adjusted OR (95% CI)	*p*-Value
COVID Group				
COVID (–)	1		1	
COVID (+)	3.503 (3.095–3.966)	<0.0001	4.25 (3.704–4.875)	<0.0001 *
Year				
2020	1		1	
2021	0.991 (0.877–1.119)	0.8807	0.697 (0.609–0.798)	<0.0001 *
Sex				
Male	1		1	
Female	0.548 (0.39–0.77)	0.0005		
Age				
<70 years	1		1	
≥70 years	3.327 (2.942–3.762)	<0.0001	2.303 (2.006–2.644)	<0.0001 *
Hospital type				
Tertiary care hospital	1		1	
General hospital	1.113 (0.976–1.27)	<0.0001	1.243 (1.08–1.43)	0.0011 *
Hospital	0.551 (0.451–0.674)	0.004	0.958 (0.774–1.187)	0.5446
Clinic	0.384 (0.276–0.535)	<0.0001	0.868 (0.614–1.227)	0.2457
Emergency surgery	2.104 (1.441–3.074)	0.0001	1.869 (1.246–2.803)	0.0025 *
ASA 3 or higher	3.601 (3.157–4.108)	<0.0001	2.115 (1.246–2.803)	<0.0001 *
Comorbidities				
History of hypertension	2.132 (1.886–2.409)	<0.0001		
History of diabetes	2.018 (1.784–2.283)	<0.0001	1.238 (1.066–1.437)	0.0052 *
History of liver disease	1.233 (1.09–1.394)	0.0008		
History of kidney disease	2.878 (2.416–3.429)	<0.0001	1.533 (1.259–1.868)	<0.0001 *
History of CVA	1.56 (0.785–3.101)	0.204		
Charson Comorbidity Index				
0	1		1	
1	1.381 (1.082–1.762)	0.0261	1.231 (0.959–1.581)	0.9933
2	1.85 (1.457–2.349)	0.0627	1.344 (1.045–1.728)	0.2247
3+	2.715 (2.243–3.286)	<0.0001	1.384 (1.103–1.737)	0.0505
Elixhauser’s comorbidities				
Weight (Continuous)	1.182 (1.16–1.205)	<0.0001		

Values are presented as odds ratios with corresponding 95% confidence intervals. * *p* < 0.05 indicates statistically significant differences between groups. OR—odds ratio; CI—confidence interval; ASA—American Society of Anesthesiologists

## Data Availability

The datasets utilized in this study are available from the corresponding author upon reasonable request. To request access, please contact Si Ra Bang at sira1045@naver.com. Data availability is subject to approval from the National Health Insurance System and adherence to the relevant permissions. As the datasets were obtained under a specific license, their use is restricted. Further information on data access procedures can be found on the National Health Insurance Sharing Service website (https://nhiss.nhis.or.kr/bd/ab/bdaba000eng.do, accessed on 1 February 2025).
